# Arthropod Assemblages in Desert Ecosystems of Alula County, Saudi Arabia

**DOI:** 10.1002/ece3.73418

**Published:** 2026-04-08

**Authors:** Ruan van Mazijk, Steven McGregor, Robbert Duker, William Liversage, Carly Butynski, Maurice Schutgens, Miren Schleicher, Max D. Graham, Shauna K. Rees, Abdelsamad Aldabaa, Ahmed H. Mohamed, Sami D. Almalki, Benjamin P. Y.‐H. Lee

**Affiliations:** ^1^ Space for Giants Nanyuki Kenya; ^2^ Department of Zoology Nelson Mandela University Gqeberha South Africa; ^3^ Wildlife and Natural Heritage Royal Commission for AlUla AlUla Saudi Arabia; ^4^ Pedology Department Desert Research Center Cairo Egypt; ^5^ Plant Ecology and Rangeland Management Desert Research Center Cairo Egypt

**Keywords:** biodiversity inventory, dryland ecology, insects, invertebrate monitoring, taxonomy, the Hejaz

## Abstract

Despite their ecological importance, epigeic arthropod assemblages in hyper‐arid systems remain understudied, particularly across the Arabian Peninsula. We provide an initial description of epigeic arthropod assemblages sampled in two protected areas in AlUla County in the Kingdom of Saudi Arabia, comparing arid thorn woodland and *wadi* habitats. Standardised pitfall trapping was done across eight sites during a single 6‐day sampling period in spring 2024. Arthropod assemblages differed between habitat types in abundance, diversity and evenness, while higher‐level taxonomic composition was broadly similar. Arid thorn woodlands tended to support greater arthropod abundances, largely driven by ants (order Hymenoptera: family Formicidae), whereas *wadis* supported more even and morphospecies‐diverse assemblages. Springtails (class Collembola: specifically order Entomobryomorpha) accounted for 24% of all individuals sampled and were recorded across both habitat types despite extreme aridity. We also report the first local records, to our knowledge, of the spider family Tetragnathidae in AlUla County, based on three morphospecies. These findings provide an initial empirical reference for a poorly studied hyper‐arid region and highlight the need for expanded temporal sampling, broader habitat coverage and improved taxonomic resolution to better characterise arthropod assemblage patterns and their potential relevance for monitoring and management.

## Graphical abstract

This study provides preliminary descriptions of arthropod communities in two desert habitats—arid thorn woodlands and *wadis*—across protected areas in AlUla County, in the Kingdom of Saudi Arabia. Pitfall trapping over a 6‐day period yielded more than 2000 specimens representing at least 189 morphospecies, with ants (Formicidae) and springtails (Collembola) dominant across both habitats. Arthropod diversity tended to be greater in *wadis* than in arid thorn woodlands, and observations included records of Tetragnathidae spiders not previously documented in the county.

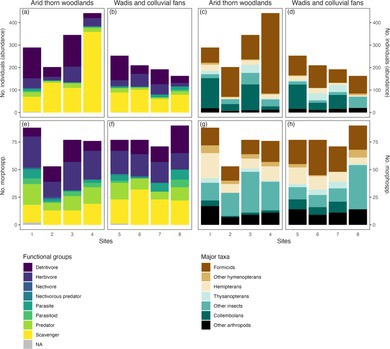

## 1 Introduction

Arthropods comprise most terrestrial faunal diversity (Ødegaard 2000; Stork 2018; Rosenberg et al. 2023) and contribute to ecosystem functioning through their roles in nutrient cycling, pollination, soil processes and food webs (Wong et al. 2019; Shehzad et al. 2024; Cardoso et al. 2025). Despite this, arthropod assemblages remain understudied in hyper‐arid regions in comparison to more mesic environments (Davidson and Groner 2021; Conkey et al. 2023). This is particularly evident across the hyper‐arid Arabian Peninsula, where ecological studies have predominantly focused on vertebrates—including mammals (Mallon et al. 2023; Al‐Musfir and Yamaguchi 2008) and birds (Alatawi et al. 2018; Mohedano et al. 2014).

Hyper‐arid landscapes are characterised by extreme climatic conditions with sparse, patchily distributed vegetation and resources (Eckardt et al. 2022). Within these landscapes, geomorphological features associated with water redistribution, such as episodic fluvial systems or ‘*wadis*’ create localised variation in both substrate and microclimate conditions relative to surrounding habitat types (Valorhiz and IUCN 2024). Evidence from other dryland regions indicates that such habitat contrasts can influence the distribution and activity of epigeic arthropods (defined here as arthropods active above the soil surface; Landsman and Thiel 2021). Empirical data from the hyper‐arid Arabian Peninsula remain limited, however, and are largely restricted to a small number of case studies (e.g., Hammad et al. 1982; Al‐Khalifa and Bayoumi 1983; Alsaleem et al. 2024; Simone et al. 2024; Alzahrani et al. 2025) or to taxon‐specific investigations, including descriptive work on ants (Sharaf et al. 2018) and termites (Sharaf et al. 2021).

This study provides initial descriptions of epigeic arthropod assemblages sampled in two protected areas in hyper‐arid AlUla County in the northwestern Kingdom of Saudi Arabia (KSA). Arthropod abundance, morphospecies composition and functional group structure were compared between two habitat types, namely arid thorn woodlands and *wadis* and colluvial fans (hereafter ‘*wadis*’)—classified by Valorhiz and IUCN (2024)—using standardised pitfall trapping during a single 6‐day sampling event in spring 2024. These findings provide preliminary insights into a poorly documented region in terms of epigeic arthropod assemblages and support future seasonal, taxonomic and restoration‐oriented research in the region.

## 2 Methods

### 2.1 Study Sites

AlUla County (26°36′11.30″ N; 37°55′46.14″ E) in northwestern KSA is hyper‐arid, receiving < 100 mm average annual rainfall (Hasanean and Mansour 2015) with daily average high temperatures of 21°C–38°C (WeatherSpark 2025). The soils throughout the county are predominantly well‐drained, nutrient‐poor sands (Maurice et al. 2023; Robin‐Soriano et al. 2024) with limited organic carbon stocks (McGregor et al. 2026) and substantial susceptibility to erosion (Amin 2004). The plant communities here are comprised of patchily distributed hyper‐arid‐adapted vegetation—predominantly woody shrubs and trees—with short‐lived herbaceous plants emerging after rains (McGregor et al. 2025).

**FIGURE 1:**
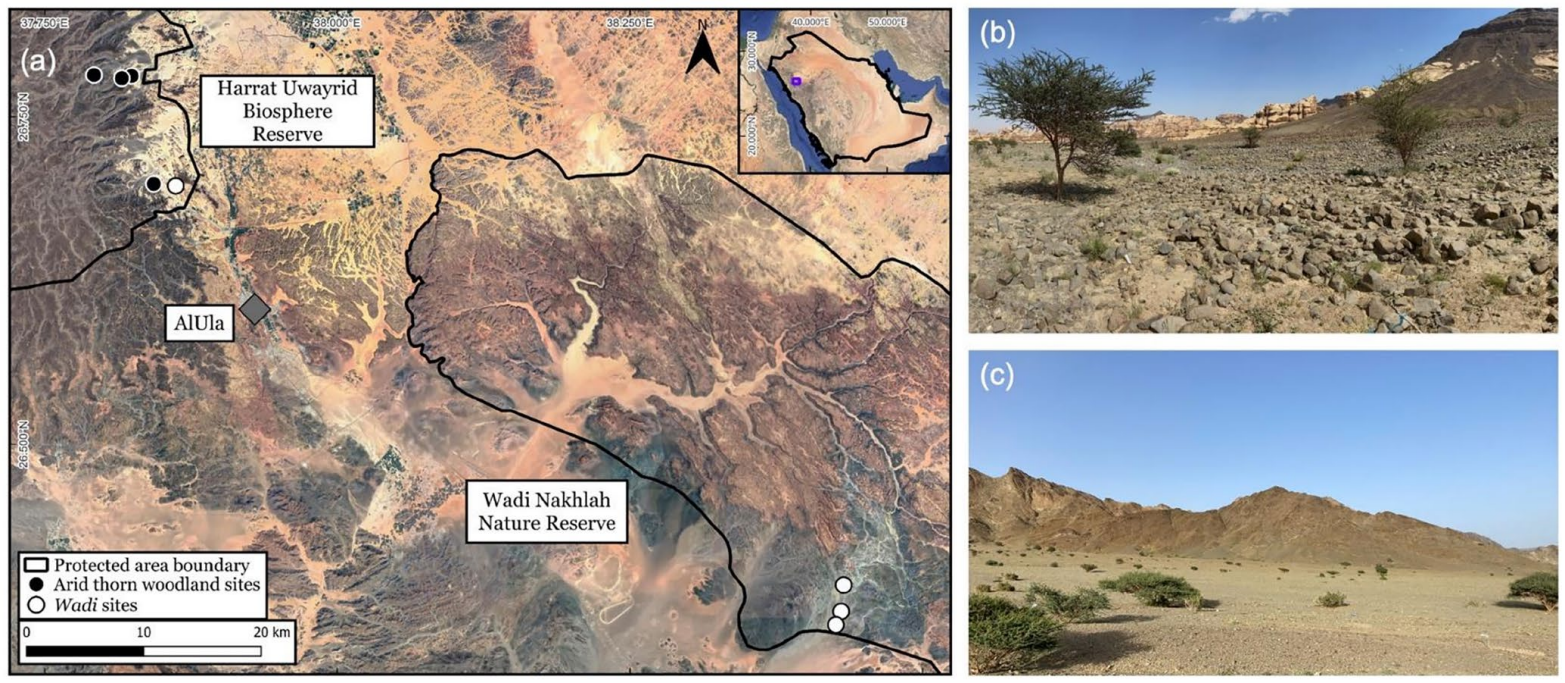
(a) Locations of the two protected areas (black polygons) sampled in AlUla County, Saudi Arabia, with sampled sites indicated and exemplified: (b) arid thorn woodlands (*n* = 4; black points in a); and (c) *wadis* (*n* = 4; white points in a).

Sampling was done in two protected areas in AlUla County: Harrat Uwayrid Biosphere Reserve (area = 468,013 ha) and Wadi Nakhlah Nature Reserve (area = 242,655 ha; Figure 1a). Within each protected area, epigeic arthropods were sampled across two habitat types—arid thorn woodlands (Figure 1b) and *wadis* (Figure 1c)—following the habitat classification of Valorhiz and IUCN (2024). Arid thorn woodlands are typically sheltered by sandstone buttes and canyons, characterised by *Vachellia* spp. (family Fabaceae) and other woody vegetation, particularly shrubs, while *wadis* are episodic fluvial systems with sparse woody vegetation (Valorhiz and IUCN 2024).

### 2.2 Field Sampling

Samples were collected in spring (1–6 May 2024), under a permit issued by the Royal Commission for AlUla (RCU) to collect arthropods within the two protected areas (permit number: WNH‐R&A_CARBON_03‐06‐2024). In arid thorn woodland sites (*n* = 4) and *wadi* sites (*n* = 4; Figure 1a), epigeic arthropods were sampled using pitfall traps arranged in a ~20‐m diameter plot (paired with eight of the randomly selected plots from a parallel study: McGregor et al. 2026). In each sampling plot, 10 pitfall traps were set, each being a 12‐cm deep (8‐cm diameter) plastic jar and containing a diluted soapy water solution following Whitman et al. (2019). Traps were placed ≥ 2‐m apart in holes dug such that the trap rims were flush with, or slightly below, the ground surface. Traps were located both in exposed sandy patches and near vegetation to reflect local variation in ground cover. Pitfall traps were left out overnight, typically for ~16 h. Specimens collected from the 10 traps set at each site were pooled to form a single composite sample per site and preserved in ethanol (Whitman et al. 2019). These composite samples (*n* = 4 per habitat type) were transported to the laboratory and used in all subsequent analyses. The full complement of specimens collected are housed with RCU.

### 2.3 Laboratory Analyses

In the laboratory, specimens were sorted and counted under a stereomicroscope using forceps. These specimens were identified to order level and (where morphological evidence was sufficient) to superfamily or family level, using morphological traits typical of these relatively stable higher taxa, without reliance on a single diagnostic key (but with input from broad entomological reference texts; e.g., Scholtz and Holm 2008). In addition, individual specimens were assigned to morphospecies based on unique external characteristics—including body size, colouration, hairs, leg morphology, antennae morphology, wing venation, petiole morphology (for ants; order Hymenoptera: Formicidae), and jaw, mandible or chelicerae (for arachnids) morphology. Specimens were also classified into eight functional groups based on morphology indicative of diet (e.g., mandible morphology) and the ecology and functional information of higher taxa—namely detritivore, herbivore, nectivore, nectivorous predator, parasite, parasitoid, predator and scavenger.

### 2.4 Data Analyses

The counts of arthropod individuals, morphospecies, superfamilies or families, orders and functional groups in each habitat were totalled and plotted using the ‘tidyverse’ suite of packages (v2.0.0; Wickham et al. 2019) in R (v4.3.1; R Core Team 2023). Diversity indices were calculated for each site using the ‘diversity’ function in the R package ‘vegan’ (v2.6‐4; Oksanen et al. 2022), namely the Shannon index (*H′* = −∑ *p*_*i*_ ln[*p*_*i*_], where *p*_i_ is the relative abundance of the *i* ^th^ species; Shannon 1948) and Pielou's evenness (*J* = *H′* ÷ ln[*H′*]; Pielou 1966). These indices were calculated with arthropod abundances (i.e., individual counts) grouped both by morphospecies and functional groups.

Since ants (Hymenoptera: Formicidae) often dominate pitfall trap samples and differ markedly in life history from most other arthropods, assemblage analyses were repeated with Formicidae excluded to assess whether patterns were driven primarily by ant dominance (Landsman and Thiel 2021). Given the high abundance of Collembola in the samples, additional analyses excluding Collembola were also done to evaluate their influence on assemblage‐level diversity patterns.

Observed differences in abundance, diversity and morphospecies richness between the two habitat types sampled were assessed with two‐sided *t*‐test (Student 1908). Prior to these analyses, Shapiro–Wilk tests were used to assess normality and *F*‐test to assess homogeneity of variances. Where these assumptions were violated, two‐sided Mann–Whitney *U*‐test were used to confirm whether the same qualitative conclusions were obtained. Given the limited number of sites sampled (*n* = 4 per habitat type), these statistical tests are interpreted primarily as indicators of patterns or trends rather than definitive tests of significance.

For the total morphospecies richness pooled across all samples, we accounted for sample size and incomplete sampling with the bias‐corrected Chao's estimator (O'Hara 2005; Chiu et al. 2014) using the ‘estimateR’ function in ‘vegan’ (number of bootstrap samples = 100). This extrapolation represents the likely lower bound of morphospecies richness. To further assess richness patterns between habitat types while accounting for sampling completeness, we compared these Chao's estimator values between habitat types. We deliberately do not compare rarefied estimates of morphospecies richness corrected to a common level of abundance (i.e., sampling effort), as we wished to investigate, not ameliorate, variation in the abundance of individual arthropods at these sites. Moreover, sampling completeness itself was estimated using iNEXT (Chao et al. 2014, 2016), for morphospecies richness (i.e., Hill number order *q* = 0) across all samples (also with number of bootstrap samples = 100).

## 3 Results

**FIGURE 2:**
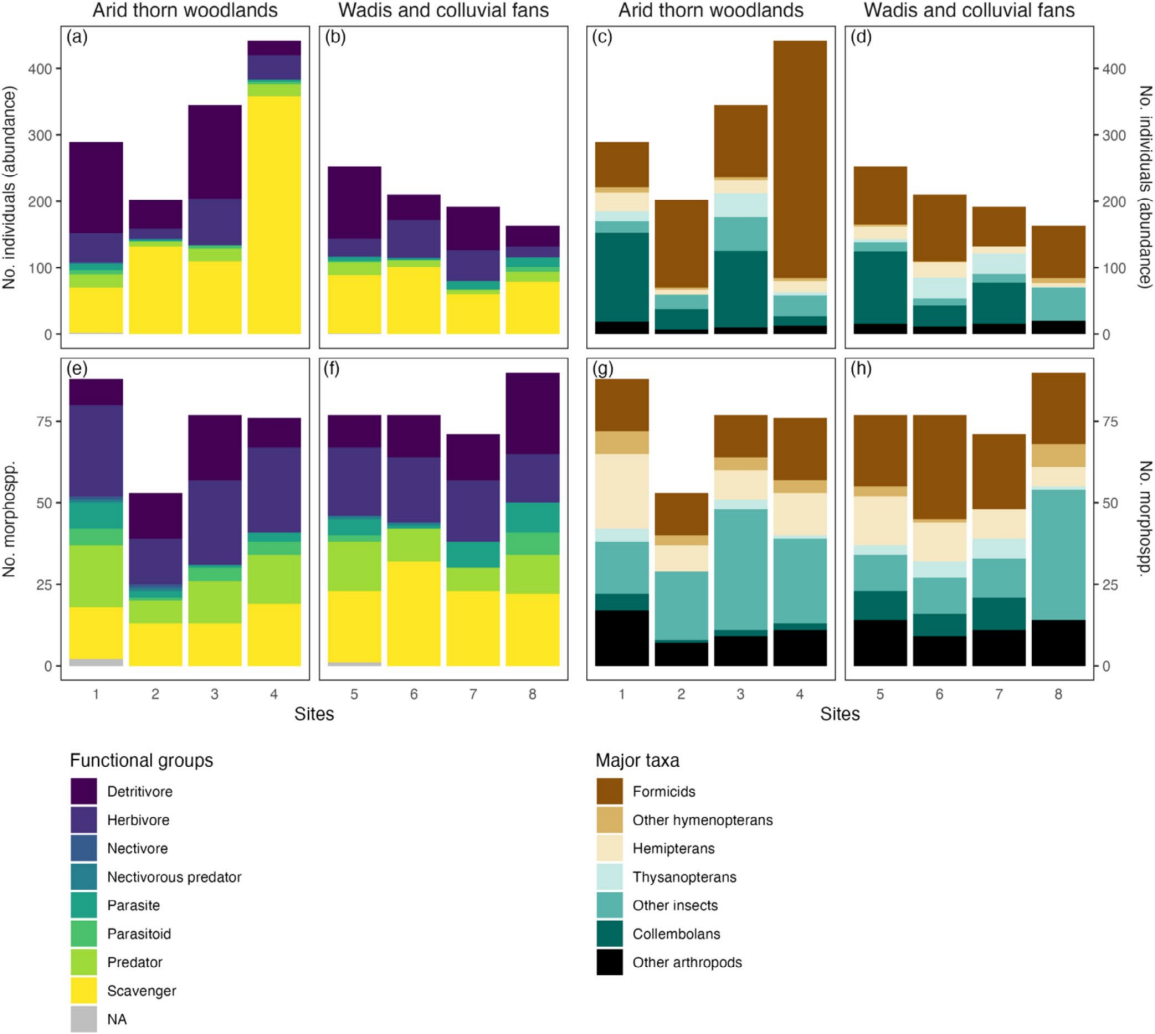
Arthropod (a–d) abundance and (e–h) morphospecies richness sampled at each of the arid thorn woodland (*n* = 4) and *wadi* sites (*n* = 4). Totals are subdivided and coloured by (a, b, e, f) functional group and (c, d, g, h) major taxa.

**FIGURE 3:**
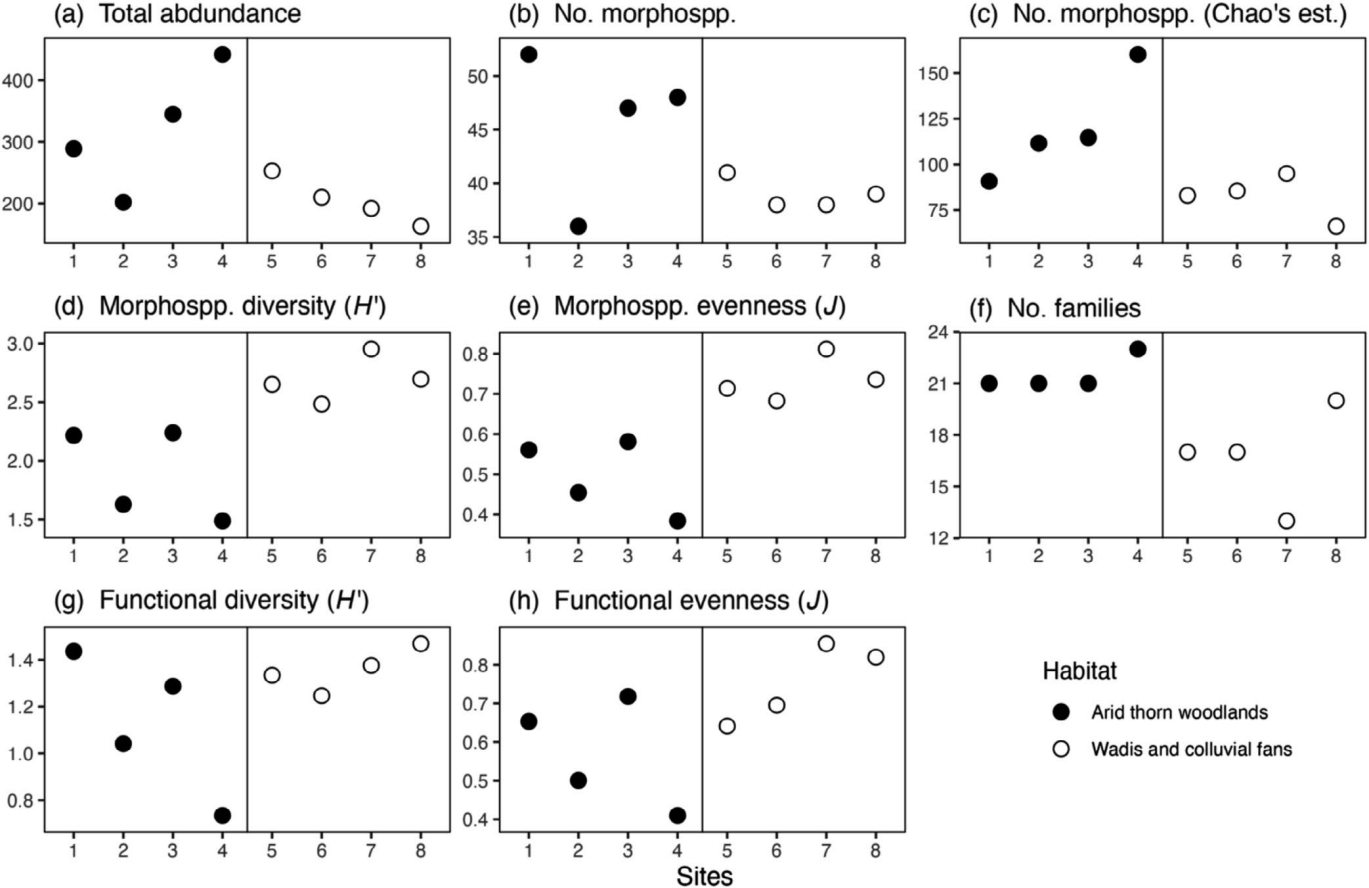
Arthropod assemblage statistics for each of the arid thorn woodland (*n* = 4; filled points) and *wadi* sites (*n* = 4; unfilled points). Each point represents the value of each statistic for one site, namely: (a) total abundance, (b) morphospecies richness, (c) Chao's estimator of morphospecies richness, (d) morphospecies diversity (Shannon's *H'*), (e) morphospecies evenness (Pielou's *J*), (f) family richness, (g) functional group diversity (*H'*), and (h) functional group evenness (*J*).

Across the eight sites sampled, 2096 epigeic arthropods were recorded, representing 189 morphospecies in at least 59 families across 16 orders and at least eight functional groups (Table 1). The extrapolated morphospecies richness across these sites was 344.0 (±SE of 45.5), with a sampling completeness of 64.8% (95% CI: 59.9%–69.8%). Arthropod assemblage composition varied among the eight sites sampled (Figures 2 and 3), and patterns in abundance, diversity and evenness differed between arid thorn woodland and *wadi* habitats. All comparisons below result in the same qualitative conclusion when using either two‐sided *t*‐test or Mann–Whitney *U*‐test; the *p*‐values presented are for these *t*‐test, unless otherwise stated.

**TABLE 1:** Total counts of morphospecies, individual specimens (*n*; i.e., abundance) and functional groups across taxonomic levels (classes, orders, superfamilies and families) for the arthropod communities sampled in our study.

Class	Order or no. orders	Superfamily, family or no. thereof	No. morphospp.	*n*	*n*/*n*_total_ (%)	*n*/*n*_order_ (%)	Functional group or no. functional groups
All classes	16	≥ 59	189	2096	100		≥ 8
Arachnida	6	≥ 16	45	110	5.2		2
	Araneae	≥ 11	27	50	2.4		All predators
		Gnaphosidae	1	1		2	
		Linyphiidae	1	5		10	
		Lycosidae	1	9		18	
		Miturgidae	1	1		2	
		Oecobiidae	1	2		4	
		Salticidae	3	3		6	
		Selenopidae	1	1		2	
		Sparassidae	5	13		26	
		Tetragnathidae	3	5		10	
		Theridiidae	2	2		4	
		Thomisidae	2	2		4	
		—	6	6		12	
	Ixodida	Ixodidae	2	7			Parasite
	Opiliones	—	1	3			Predator
	Scorpiones	Buthidae	2	2			Predator
	Solifugae	—	2	2			Predator
	Trombidiformes	—	11	46	2.2		Parasite
Collembola	Entomobryomorpha	—	25	496	24		Detritivore
Crustacea	Isopoda	—	1	1			Detritivore
Insecta	8	≥ 40	118	1489	71		≥ 7
	Coleoptera	≥ 11	21	74	3.5		≥ 3
		Anthicidae	3	20	1.0	27	Predator
		Buprestidae	1	1		1.4	Herbivore
		Carabidae	3	30	1.4	41	Predator
		Chrysomelidae	2	3		4.1	Herbivore
		Coccinellidae	1	1		1.4	Predator
		Curculionidae	1	1		1.4	Herbivore
		Elateridae	1	2		2.7	Herbivore
		Scarabaeidae	1	1		1.4	Herbivore
		Silvanidae	1	1		1.4	Herbivore
		Staphylinidae	3	4		5.4	Predator
		Tenebrionidae	3	9		12	Detritivore
		—	1	1		1.4	Unknown
	Diptera	≥ 6	17	86	4.1		3
		Empidoidea	1	1		1.2	Predator
		Ephydroidea	2	7		8.1	Detritivore
		Muscidae	7	16		19	Detritivore
		Phoridae	2	39	1.9	45	Detritivore
		Sciaroidea	3	17		20	Detritivore
		Tephritidae	1	5		5.8	Herbivore
		—	1	1		1.2	Detritivore
	Hemiptera	≥ 10	32	132	6.2		2
		Aphididae	1	3		2.3	Herbivore
		Cicadellidae	13	97	4.6	74	Herbivore
		Lygaeidae	2	3		2.3	Herbivore
		Miridae	3	5		3.8	Herbivore
		Nabidae	1	1			Predator
		Pentatomidae	1	3		2.3	Herbivore
		Psyllidae	4	9		6.8	Herbivore
		Reduviidae	1	2		1.5	Predator
		Tingidae	1	1			Herbivore
		Tropiduchidae	1	1			Herbivore
		—	4	7		5.3	Herbivore
	Hymenoptera	5	28	1025	53		4
		Apoidea	2	2			Nectarivore
		Chalcidoidea	17	24	1.1		Parasitoid
		Crabronidae	2	2			Nectivorous predator
		Formicidae	6	995	47	97	Scavenger
		Pompilidae	1	2			Nectivorous predator
	Lepidoptera	—	10	27	1.3		Herbivore
	Orthoptera	2	6	21	1.0		All herbivores
		Acrididae	5	10		48	
		Gryllidae	1	11		52	
	Thysanoptera	—	3	123	5.9		Herbivore
	Zygentoma	Lepismatidae	1	1			Detritivore

*Note:* Specimens unidentifiable to the superfamily and family levels are grouped with a dash (—). For *n*/*n*_total_, percentages (%) of < 1 are omitted; for *n*/*n*_order_, percentages of 100 or those not applicable are omitted.

### 3.1 Arthropod Abundance

**FIGURE 4:**
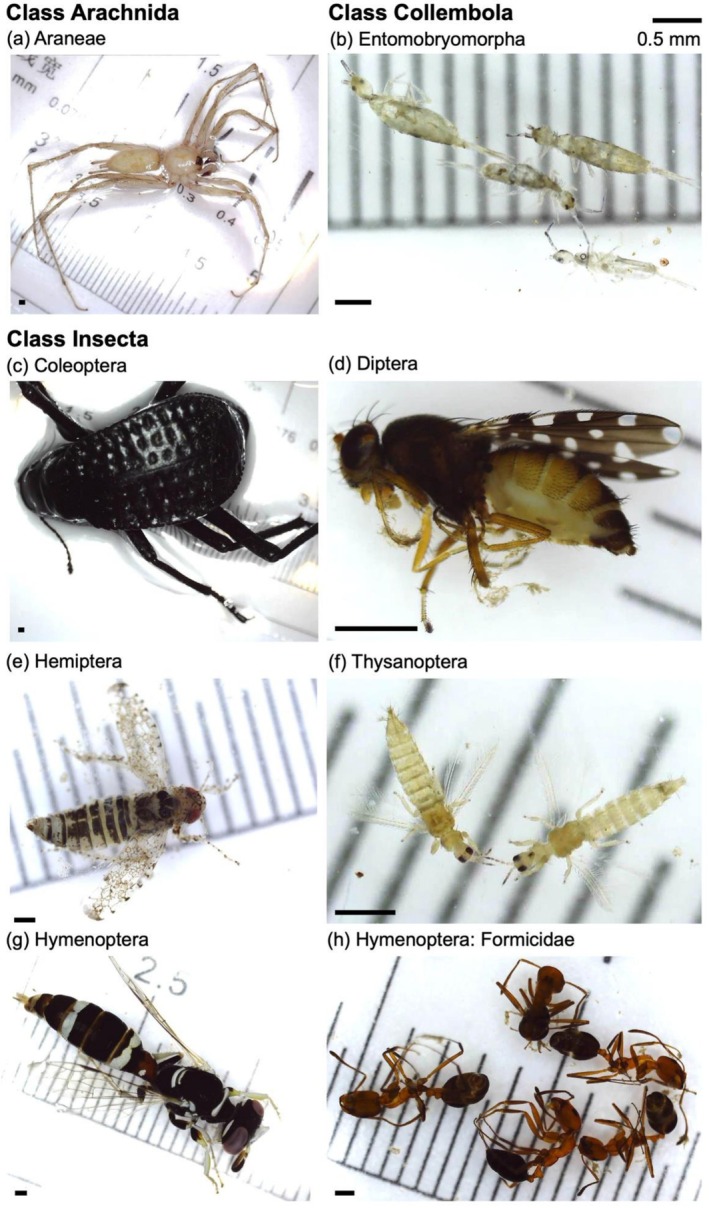
Examples of specimens belonging to dominant orders (*n* ≥ 50) collected from this study's sites in AlUla County. All scale bars (lower left of each panel) are 0.5 mm in length, as keyed. Dominant orders presented are: (a) Araneae (class Arachnida), (b) Entomobryomorpha (class Collembola), (c) Coleoptera, (d) Diptera, (e) Hemiptera, (f) Thysanoptera, and (g,h) Hymenoptera (non‐formicid and formicid representatives, respectively). Orders in panels c–h belong to class Insecta.

Arthropod abundance tended to be greater in arid thorn woodland sites (mean = 319.5 individuals) than in *wadi* sites (mean = 204.5 individuals per site; *p* = 0.10; Table 2a, Figure 3a). Across all samples and in each habitat type, the most abundant order was Hymenoptera (class Insecta; Table 1; Figure 2c,d). Within this order, the family Formicidae (ants) dominated (see Figure 4h), representing 97% of hymenopteran individuals, 67% of insect individuals and 47% of all arthropods sampled (52% in arid thorn woodlands; but 40% in *wadis*), respectively (Table 1; Figure 2c,d).

**TABLE 2:** Arthropod assemblage statistics averaged each for the arid thorn woodland (*n* = 4) and *wadi* sites (*n* = 4), respectively.

Assemblage statistic	Arid thorn woodlands	*Wadis*	*p*
(a) Total abundance	319.5	204.5	0.102
(b) No. morphospp.^a^	45.8	39	0.141
(c) No. morphospp. (Chao's est.)	119.3	82.4	0.08
(d) Morphospp. diversity (*H′*)	1.9	2.7	0.017
(e) Morphospp. evenness (*J*)	0.5	0.7	0.006
(f) No. families^b^	21.5	16.8	0.039
(g) Functional diversity (*H′*)	1.1	1.4	0.23
(h) Functional evenness (*J*)	0.6	0.8	0.084
(i) No. functional groups	7.3	6.3	0.347

*Note:* The *p*‐values presented follow two‐sided *t*‐test between the habitat types.

^a^ Morphospecies richness (b) was the only data for which there was evidence of unequal variances between arid thorn woodland and *wadi* site values (*F*_3,3_ = 23.5, *p* = 0.0277). Using either parametric or non‐parametric tests, though, yielded little evidence of differences between arid thorn woodland and *wadi* sites (*t*‐test *p* = 0.141; Mann–Whitney *U*‐test *p* = 0.309).

^b^ Family richness (f) in arid thorn woodlands was the only subset of data for which there was evidence of non‐normality (Shapiro–Wilk test, *p* = 0.001); all other data (within each habitat type and when habitats were combined) were normally distributed (Shapiro–Wilk tests, all *p* > 0.009). Both parametric and non‐parametric tests demonstrated differences in this metric between habitat types (*t*‐test *p* = 0.039, Mann Whitney *U*‐test *p* = 0.025).

The second most abundant order across both habitat types was Entomobryomorpha (class Collembola; Figure 2c,d; see also Figure 4b). Among insects, Hemiptera was the second most abundant order across all samples (Table 1), attributed primarily to the family Cicadellidae (see Figure 4e), which represented 73.5% of hemipteran individuals. Hemiptera were also the second most abundant insect order in arid thorn woodland sites (*n* = 73, 5.7% of individuals), whereas Thysanoptera were the second most abundant insect order in *wadis* (*n* = 67, 8.1% of individuals; Figure 2c,d; see also Figure 4f).

### 3.2 Morphospecies Diversity Across Habitats

Observed arthropod morphospecies richness may be similar between arid thorn woodland and *wadi* sites (*p* = 0.14; Table 2b, Figure 3b). However, when accounting for incomplete sampling using Chao's richness estimator, morphospecies richness tended to be higher in arid thorn woodland sites than in *wadi* sites (mean = 119.0 vs. 82.4; *p* = 0.080; Table 2c, Figure 3c). In contrast, diversity and evenness tended to be greater in *wadi* sites than in arid thorn woodland sites. Mean Shannon diversity (*H* *′*) was 2.7 in *wadi* sites compared with 1.9 in arid thorn woodlands (*p* = 0.02; Table 2d, Figure 3d), while Pielou's evenness (*J*) averaged 0.7 in *wadi* sites and 0.5 in arid thorn woodland sites (*p* = 0.007; Table 2e, Figure 3e). Arthropod family richness also tended to be higher in arid thorn woodland sites (mean = 21.5) than in *wadis* (mean = 16.8; *p* = 0.04; Table 2f, Figure 3f).

Assemblage differences were partly associated with dominant taxa. Ant assemblages tended to be more morphospecies‐diverse in *wadis* (mean *H* *′* = 2.7, range = 2.6–2.8) than in arid thorn woodlands (mean *H′* = 1.9, range = 1.3–2.1; *p* = 0.02; Table 3). Collembola assemblages likewise tended to be more morphospecies‐diverse in *wadis* (mean *H′* = 2.0, range = 1.9–2.2) than in arid thorn woodlands (mean *H′* = 0.5, range = 0.0–0.8; *p* = 0.001; Table 3). When dominant taxa were excluded from analyses, assemblage‐level diversity tended to be similar between habitat types (excluding Formicidae: *p* = 0.34; excluding Collembola: *p* = 0.16; Table 3). In both cases, morphospecies richness tended to be higher in arid thorn woodlands than in *wadis* (excluding Formicidae: mean = 30.8 vs. 22.0, *p* = 0.08; excluding Collembola: mean = 44.8 vs. 32.8, *p* = 0.03; Table 3)—though this tendency was not evident in corresponding Chao's estimators of morphospecies richness (excluding Formicidae: mean = 526 vs. 214, *p* = 0.38; excluding Collembola: mean = 505.7 vs. 439.0, *p* = 0.596; Table 3).

**TABLE 3:** Arthropod assemblage statistics for taxonomic subsets (as indicated) of the data from this study.

Assemblage statistic	Excl. Collembola			Excl. Formicidae			Only Collembola			Only Formicidae		
	A	W	*p*	A	W	*p*	A	W	*p*	A	W	*p*
(a) Total abundance	246	154	0.237	53.5	43.5	0.482	73.5	67.3	0.875	166.8	82	0.284
(b) No. morphospp.	44.8	32.8	0.032	30.8	22	0.081	1	8.3	0.014	4	4.8	0.178
(c) No. morphospp. (Chao's est.)	505.7	439	0.596	526	214	0.384	12	12	1	229.3	168.4	0.568
(d) Morphospp. diversity (*H′*)	3.1	3.8	0.164	3.6	3.3	0.336	0.5	2	0.001	1.9	2.7	0.015

*Note:* As with Table 2, these are averaged each for the arid thorn woodland (*n* = 4, ‘A’) and *wadi* sites (*n* = 4, ‘W’), respectively. The *p*‐values presented follow two‐sided *t*‐test between the habitat types for each taxonomic subset.

### 3.3 Taxonomic Composition

**FIGURE 5:**
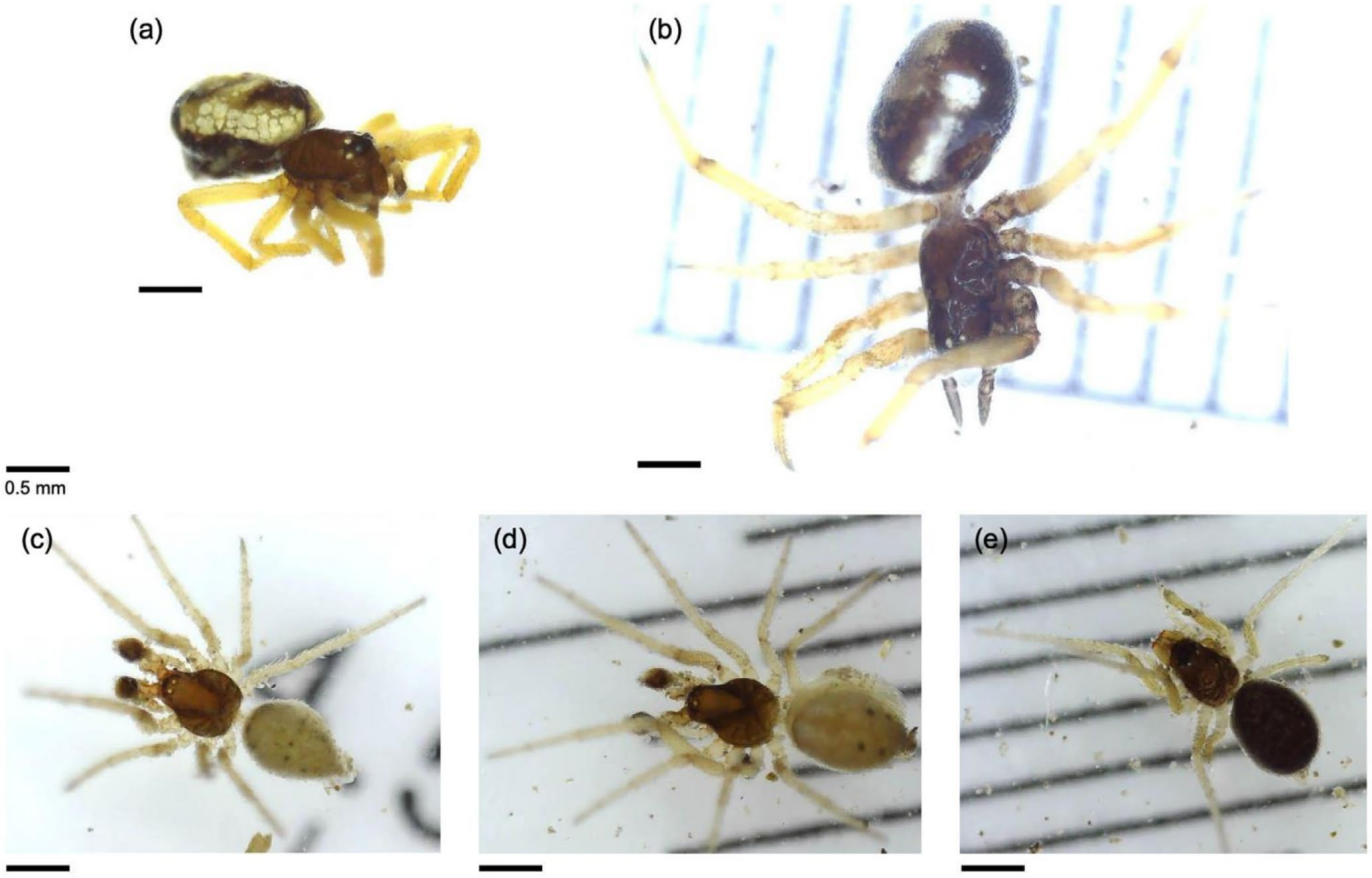
Specimens belonging to the spider family Tetragnathidae (order Araneae) collected from this study's sites in AlUla County, belonging to three morphospecies: (a, b) Tetragnathidae MS 1; (c, d) Tetragnathidae MS 2; and (e) Tetragnathidae MS 3. All scale bars (lower left of each panel) are 0.5 mm in length, as keyed. Specimens in a–c were collected from *wadi* sites, and d and e from arid thorn woodland sites.

Across all samples and within each habitat type, the most morphospecies‐rich insect order was Hemiptera (Figure 2g,h; see also Figure 4e). In *wadis*, this was exceeded by a non‐insect order, Entomobryomorpha (i.e., springtails; Figure 2h; see also Figure 4b). The next most morphospecies‐rich non‐insect order was Araneae (i.e., spiders; see Figure 4a), with 27 morphospecies recorded, predominantly within the families Sparassidae (5 morphospecies), Salticidae (3 morphospecies) and Tetragnathidae (3 morphospecies; see Figure 5; Table 1). Across all samples, spiders accounted for 13% of all morphospecies observed.

### 3.4 Functional Groups

Across all samples, the most abundant functional group comprised scavengers (all ants), followed by detritivores (Figure 2a,b). Herbivores were the most morphospecies‐rich functional group across all samples and in arid thorn woodlands (57 and 44 morphospecies, respectively), whereas detritivores were most morphospecies‐rich in *wadis* (34 morphospecies; Figure 2e,f).

The orders represented by the most observed number of functional groups were Coleoptera (4 functional groups across all samples; 3 in each habitat type) and Hymenoptera (4 functional groups across all samples and in arid thorn woodlands; 3 in *wadis*). While arid thorn woodland and *wadi* sites had similar diversities of functional groups, the functional group composition tended to show greater evenness in the *wadis* (mean *H′* = 1.1 vs. 1.4, *p* = 0.23, Figure 3g; mean *J* = 0.57 vs. 0.75, *p* = 0.08; Figure 3h).

## 4 Discussion

This study provides an initial description of epigeic arthropod assemblages of two protected areas in hyper‐arid AlUla County, KSA, based on specimens identified primarily to order, family, or morphospecies level. Comparisons between arid thorn woodland and *wadi* habitats indicate differences in arthropod abundance, diversity and evenness, although taxonomic composition was broadly similar. Arid thorn woodland sites tended to support greater arthropod abundances, predominantly of ants, whereas *wadi* sites had more even and morphospecies‐diverse assemblages.

Ants (order Hymenoptera: family Formicidae; e.g., Figure 4h) dominated samples from both habitat types and accounted for nearly half of all individuals sampled. Ants are commonly treated separately from other hymenopterans in arthropod community studies because their eusocial life history, colony‐based organisation and foraging behaviour differ markedly from those of most solitary or less social arthropod taxa (Landsman and Thiel 2021). The numerical dominance of ants observed here is consistent with their global prevalence in terrestrial ecosystems relative to other arthropod taxa (e.g., Oi 2008; Schultheiss et al. 2022) and with the tendency for pitfall traps to capture multiple individuals from the same nearby colonies (Hacala et al. 2021). Despite their numerical dominance, ant morphospecies diversity tended to be greater in *wadis* than in arid thorn woodlands. When ants were excluded from analyses, differences in morphospecies diversity between habitat types were reduced, indicating that formicid assemblages contributed substantially to the assemblage‐level contrasts observed. As central‐place foragers (Dejean et al. 2025), ant communities are often sensitive to environmental heterogeneity and resource distribution, supporting their frequent use as indicators of ecosystem processes and functioning (Tiede et al. 2017).

Springtails (class Collembola; e.g., Figure 4b) represented a large proportion of individuals sampled across sites. Their observed abundance and diversity, particularly in *wadis*, is notable given the extreme aridity and sandy soils characteristic of the AlUla region. Although collembolans are commonly associated with moist, organic‐rich substrates (Potapov et al. 2017; Krediet et al. 2023), desiccation‐tolerant taxa are known from desert and dryland ecosystems (Liu et al. 2021; Escribano‐Álvarez et al. 2024). All collembolans recorded belonged to Entomobryomorpha, an order that includes species with documented desiccation‐tolerance (e.g., Kærsgaard et al. 2004; Liu et al. 2021; Escribano‐Álvarez et al. 2024). Further taxonomic resolution and paired environmental data would be required to assess whether the collembolan assemblages observed here represent persistent populations or transient responses to favourable conditions, particularly given that sampling occurred less than a month after rainfall in the study area.

The detection of the spider family Tetragnathidae (order Araneae) in AlUla County represents, to our knowledge, the first local record (Figure 5). Although spiders are among the better‐documented arthropod groups in the region, with numerous families and species recorded across both Harrat Uwayrid Biosphere Reserve and Wadi Nakhlah Nature Reserve (see Simone et al. 2024), the AlUla invertebrate inventory does not list Tetragnathidae among the families documented to date. This highlights the continued incompleteness of arthropod records at the sub‐national scale on the Arabian Peninsula. Additional sampling with improved taxonomic resolution, including comparison with museum collections, would be required to refine the distribution and status of spider (and other arthropod) taxa within the region.

In this study, all sampling was done during spring, when arthropod activity and detectability are typically elevated due to increased moisture and food availability (Alsaleem et al. 2024; Alzahrani et al. 2025). The assemblage patterns reported here therefore likely reflect favourable seasonal conditions associated with recent rainfall. Year‐round or multi‐season sampling would be required to characterise temporal variation in these assemblages, particularly with respect to habitat‐specific responses to episodic water availability in environments such as *wadis* (Şen 2008). In addition, sampling was restricted to a single 6‐day period, and the findings reported here therefore represent a short‐term observation rather than a detailed representation of longer‐term epigeic arthropod assemblage structure (Case in point: observed morphospecies richness was seemingly similar in arid thorn woodland and *wadi* sites, yet Chao's bias‐corrected estimator thereof showed more evidence of difference between the habitats). Pitfall trapping further biases sampling towards epigeic taxa and under‐represents belowground or less mobile arthropods (Hohbein and Conway 2018). In this context, the notable absence of termites from our samples may reflect limited representation of aboveground‐foraging termite species in the study area, low sampling effort, or conditions that were unfavourable for aboveground termite activity, despite their known regional presence (Sharaf et al. 2021).

This study documents distinct patterns in epigeic arthropod assemblages across arid thorn woodland and *wadi* habitats in AlUla County, KSA—as defined by the habitat classification of Valorhiz and IUCN (2024)—and identifies ants and collembolans as prominent contributors to observed differences in assemblage structure between habitat types. The findings provide an initial empirical reference for a hyper‐arid region where arthropod data remain sparse and establish a foundation for a more systematic research programme. Although taxonomic resolution was limited to morphospecies, the habitat‐associated patterns observed, together with the strong contribution of taxa such as ants, indicate that epigeic arthropods warrant further evaluation as potential indicators in monitoring and management‐oriented studies, contingent on finer taxonomic resolution and expanded spatial coverage. Future priorities include year‐round and multi‐season sampling to characterise temporal dynamics, expanded sampling across a broader range of habitat types, the use of complementary sampling methods to capture taxa across different strata and functional groups, and improved taxonomic resolution to verify novel or poorly documented regional records. Where clear contrasts in land use or management occur, comparative sampling across adjacent land‐use boundaries would also support the assessment of arthropod assemblage responses to conservation or restoration interventions.

## Author Contributions

**Ruan van Mazijk:** conceptualization (lead), data curation (equal), formal analysis (lead), writing – original draft (equal), writing – review and editing (equal). **Steven McGregor:** conceptualization (lead), data curation (equal), writing – original draft (equal), writing – review and editing (equal). **Robbert Duker:** conceptualization (supporting), writing – review and editing (equal). **William Liversage:** conceptualization (supporting), visualization (supporting), writing – review and editing (equal). **Carly Butynski:** conceptualization (supporting), writing – review and editing (equal). **Maurice Schutgens:** conceptualization (supporting), writing – review and editing (equal). **Miren Schleicher:** conceptualization (supporting), writing – review and editing (equal). **Max D. Graham:** conceptualization (supporting), writing – review and editing (equal). **Shauna K. Rees:** conceptualization (lead), writing – review and editing (equal). **Abdelsamad Aldabaa:** conceptualization (lead), writing – review and editing (equal). **Ahmed H. Mohamed:** conceptualization (lead), writing – review and editing (equal). **Sami D. Almalki:** conceptualization (supporting), writing – review and editing (equal). **Benjamin P. Y.‐H. Lee:** conceptualization (lead), writing – review and editing (equal).

## Conflicts of Interest

The authors declare no conflicts of interest.

## Data Availability

The datasets generated and analysed for this study are open access and available here: https://doi.org/10.25408/mandela.29128043.
